# Reengineering of MeSH thesauri for term selection to optimize literature retrieval and knowledge reconstruction in support of stem cell research

**DOI:** 10.1186/s12911-016-0298-z

**Published:** 2016-05-23

**Authors:** Yan Su, James Andrews, Hong Huang, Yue Wang, Liangliang Kong, Peter Cannon, Ping Xu

**Affiliations:** Shanghai Information Center for Life Sciences, Chinese Academy of Sciences, Shanghai, China; School of Information, University of South Florida, Tampa, FL USA

**Keywords:** Knowledge organization system (KOS), MeSH, Thesaurus, Classify, Stem cells

## Abstract

**Background:**

PubMed is a widely used database for scientists to find biomedical-related literature. Due to the complexity of the selected research subject and its interdisciplinary nature, as well as the exponential growth in the number of disparate pieces of biomedical literature, it is an overwhelming challenge for scientists to define the right search strategies and quickly locate all related information. Specialized subsets and groupings of controlled vocabularies, such as Medical Subject Headings (MeSH), can enhance information retrieval in specialized domains, such as stem cell research. There is a need to develop effective search strategies and convenient solutions for knowledge organization in stem cell research. The understanding of the interrelationships between these MeSH terms also facilitates the building of knowledge organization systems in related subject fields.

**Methods:**

This study collected empirical data for MeSH-related terms from stem cell literature and developed a novel approach that uses both automation and expert-selection to create a set of terms that supports enhanced retrieval. The selected MeSH terms were reconstructed into a classified thesaurus that can guide researchers towards a successful search and knowledge organization of stem cell literature.

**Results:**

First, 4253 MeSH terms were harvested from a sample of 5527 stem cell related research papers from the PubMed database. Next, unrelated terms were filtered out based on term frequency and specificity. Precision and recall measures were used to help identify additional valuable terms, which were mostly non-MeSH terms. The study identified 15 terms that specifically referred to stem cell research for information retrieval, which would yield a higher precision (97.7 %) and recall (94.4 %) rates in comparison to other approaches. In addition, 128 root MeSH terms were selected to conduct knowledge organization of stem cell research in categories of anatomy, disease, and others.

**Conclusions:**

This study presented a novel strategy and procedure to reengineer term selections of the MeSH thesaurus for literature retrieval and knowledge organization using stem cell research as a case. It could help scientists to select their own search terms and build up a thesaurus-based knowledge organization system in interested and interdisciplinary research subject areas.

**Electronic supplementary material:**

The online version of this article (doi:10.1186/s12911-016-0298-z) contains supplementary material, which is available to authorized users.

## Background

In this “big data” era, the richness of information and the speed at which it spreads is nearly unimaginable. While this may improve convenience, it also leads to a certain disorder of both information and knowledge. This poses a challenge that can impede the effective use of research publications and related knowledge. This problem requires numerous efforts to build new strategies of information retrieval or knowledge organization in order to facilitate knowledge discovery and analysis [1].

Scientific literature is an important part of the overall universe of information resources. Quantitative analysis of this provides a basis for knowledge management that requires highly accurate information retrieval. Among the numerous challenges with achieving quality retrieval, two are particularly salient in the context of stem cell research. First, across various scientific disciplines that may be interested in stem cell research, there is variance in expressing the same concept. Thus, it is very difficult to list every synonym (or quasi-synonym) for each concept. Second, some technologies cover content in disparate knowledge fields, and therefore cannot be searched from a single perspective. For example, tissue engineering covers a broad range of scientific research, and so it is difficult to anticipate and provide for all the related search terms without creating a unique ontology or other techniques. Recognizing this as a research topic that is important not only in the context of stem cell research but in other domains, we have designed an approach to partially but effectively address the problem of literature retrieval and classification [2, 3].

At present, three databases, PubMed, Web of Knowledge, and Scopus, are widely used in biomedical informatics [4, 5]. Each of these three platforms can be called a Knowledge Organization System (KOS), another term for a classification system or, depending on its semantic structuring, a thesaurus, which allows for statistical analysis of the retrieval results [4]. Among these three platforms, only PubMed indexes literature with a standardized vocabulary, the Medical Subject Headings (MeSH). MeSH is a highly representative and widely used controlled vocabulary designed for the indexing of journal articles, books, and related artifacts in the health and life sciences [6, 7]. Within the MeSH vocabulary, semantics are clear and it has strict norms combined with logical relationships among terms. When appropriately applied, MeSH has the potential to clearly provide subject headings for the biological information being communicated through an article [8, 9]. It is widely considered the most effective controlled terminology for biomedical literature retrieval and certain types of information mining [5, 10–13]. Using controlled, organized vocabularies or thesauri, such as MeSH, enables researchers to spend less time and effort in synonym merging and polysemy splitting to ascertain relationships between terms and concepts.

As a KOS, a thesaurus can sort and reorganize knowledge according to the content and characteristics of the subject. When used in literature retrieval, a thesaurus can recognize hyponymy of a retrieved field and recognize the sophisticated classification of the retrieved data set [14, 15]. By doing so, thesauri can be helpful for solving the problem of retrieval by improving recall and precision. However, thesauri often express specific things while data analysis is broader and involves items of a similar property, otherwise known as a category. That is to say, when a category is retrieved using one or two terms from the thesaurus, it may not be enough to narrow the search enough to find appropriate results [16, 17]. Additional thesauri need to be included and merged into different categories. Furthermore, a thesaurus such as MeSH includes many terms that cover all the areas of health science, in order to manage knowledge and for effective retrieval. MeSH terms and the current hierarchical structure, therefore, might lack “specificity” to identify a precise set of thesauri to fully represent the concepts and knowledge structure in a special interdisciplinary research area [18]. This is needed for stem cell research in order to retrieve literature with satisfactory rates of precision and recall.

Although covering a broad range of topics in health sciences, the hierarchical structures of MeSH do not have the specificity required in specific technical domains such as stem cell research. This is one of the reasons why it is not ideal for patient records. However, many concepts in MeSH showed as “hierarchical tree structure” do have rather specific sub-concepts or so-called “children” (even in more than one tree), but these are not always the semantic relations or syntactic structures needed for a particular context (such as stem cell research). For example, the term “K562 Cells” resides in the lower level of “Myeloid Progenitor Cells” and is an important keyword in the field of stem cells. However, the term “K562 Cells” is related to the cell lines widely used in regular lab experiments, and it has little to do with the stem cells research. In a literature search using “Myeloid Progenitor Cells,” the system traversed the “child” terms, including “K562 Cells”, and thus the search could bring in noisy literature.

In fact, multiple, combinational search keywords were needed to cover full-specific topics, such as stem cell research. Both automated and/or human recommended MeSH terms were used for the literature search and annotation [19, 20]. Professional searchers craft combinational search queries when using MeSH to search specific subjects. The terms reside discretely or aggregate in different locations of the hierarchy structure of the MeSH “tree structure.” For example, the hyponyms of “stem cells” include “embryonic stem cells,” “induced pluripotent stem cells,” “adult stem cells” and “cancer stem cells” in the field of stem cell research. However, stem cell research covers an even wider scope, and terms such as “hematopoietic stem cell transplantation,” “stem cell microenvironment” and “transdifferentiation” are not hyponyms under “stem cells.” Therefore, only using “stem cells” to search might omit certain relevant research related literature. MeSH indexing is based on the concept level, and for stem cell research activities, such as knowledge discovery and organization, one or two MeSH terms are usually not enough to represent the complexity of stem cell research. We propose to address this by attempting to reconstruct MeSH into a classified thesaurus based on a specific perspective and context.

The stem cell area is at the cutting-edge of science research. New breakthroughs are frequently and actively reported in the literature. Some of them were listed in the top ten lists of scientific breakthroughs in Science Magazine since 1999. Stem cells, or related derivatives, can be transplanted into a patient to replace damaged cells and regenerate new cells or tissues [21]. It brings new hope for the treatment of “incurable diseases” [22]. Using stem cells for the development of disease models for drug screening and/or pinpointing disease mechanisms can help researchers understand the mechanism of pathogenesis for complex diseases [23]. Stem cell research is a rapidly changing field, and it an interdisciplinary and multi-faceted area of study. Selecting all of the relevant stem cell literature from the interconnected disciplinary based literature databases required scientists to create a better set of key words to allow for search completeness and accuracy. The exploration of stem cell related MeSH classifications could also benefit the knowledge construction and classification for further future stem cell research.

This reconstruction is the process of knowledge reorganization. If embedded into a retrieval system, the classified thesaurus will create literature category navigation and automatic classification. This helps achieve an automated literature analysis that would be useful in serving the information needs of researchers in this domain, which could be replicated for other research areas.

## Methods

**Fig. 1:**
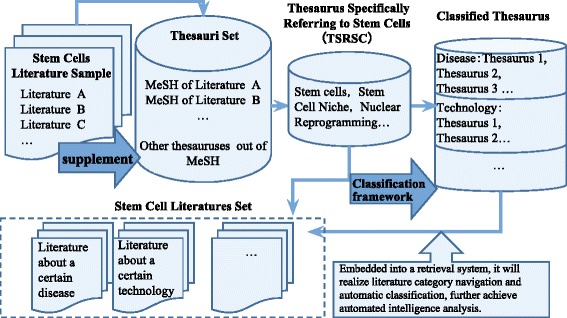
The process of MeSH terms selection and construction of the TSRSC

Figure 1 shows the technical approach used for the construction of a thesaurus specifically referring to stem cells (TSRSC), classification frameworks, and classified thesauri. This involved a term selection strategy that combined both automation and expert selection to process specially harvested MeSH terms for information retrieval and knowledge reconstruction. This method included the following steps:

1. **Construction of the TSRSC**. The MeSH vocabulary includes a variety of terms about stem cells primarily under the MeSH term “stem cells,” although there are other related terms under other term nodes. Terms for the TSRSC were identified based on MeSH term frequencies in the literature sample. In addition, stem cell research experts from Shanghai Institutes for Biological Science, Chinese Academy of Science, helped identify additional stem cell related MeSH terms that are not indexed but are otherwise important in the field. In our study, we collected the literature sample from PubMed and each stem cell research-related article was indexed with 7–8 MeSH terms. It should be noted, however, that, during this process, it is possible that some stem cell research related MeSH terms were not included. Additionally, experts helped identify other highly relevant terms that were not MeSH terms that would be included for information retrieval, improving the recall and precision ratios of the literature retrieval sets for stem cell research. The recall and precision ratios were tested and the experts were further consulted to optimize TSRSC.
2. **Classification framework**. Based on the existing classification scheme, this study developed a specific classification framework for stem cell research. Such a classification framework was built to be suitable for literature analysis of stem cells, including disease categories such as neoplasms, cardiovascular disease, and respiratory tract diseases, as well as for technology categories like reprogramming and stem cell transplantation.
3. **Classified thesaurus**. High-frequency MeSH terms from a stem cell-related subset were filtered and classified into different categories. The classification thesaurus was optimized by follow-up retrieval experiments.

**Fig. 2:**
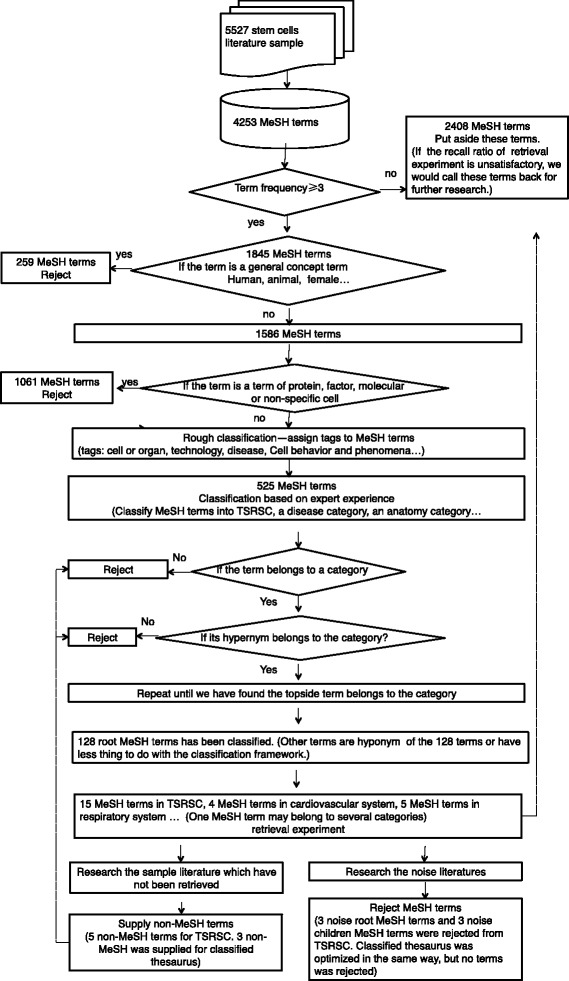
Flow Chart of the tasks to obtain thesauri specifically referring to stem cells (TSRSC)

The classified thesaurus was built following the method outlined in Fig. 2, which included:

1. A sample of stem cell literature selected from 17 stem cell specific journals. In total, 5527 stem cell-related articles were obtained from the years 2002/01/01 to 2011/12/31. The MeSH terms were extracted and their frequencies were counted. The articles were randomized into a set of 1000. It was determined that 98.6 % of the articles in the set were about stem cells, indicating that the sample was representative of stem cell research.
2. MeSH term frequency statistics. MeSH term frequencies were calculated and ranked in the literature sample. A total of 4253 MeSH terms were obtained. Their term frequencies were ranked from the highest to the lowest. A cutoff of three was selected with a subset of 1825 MeSH terms. They were further identified and classified by stem cell experts. This subset represented over 95 % of total occurrences of all MeSH terms with a good coverage for the sample of the literature.
3. Excluding unrelated keywords. Common words, such as “Human,” “Animals,” and “Cell Line” are too general and, therefore, were excluded. In addition, other MeSH terms, such as those related to protein subunits, DNA sequencing, and other chemical molecules, were removed from the list due to their broad meaning and difficulty in being classified. A total of 1320 terms were excluded while the experts retained 525 keywords for the next phase.
4. Coarse-grained classification of MeSH terms. Experts determined the remaining MeSH terms one by one, labeling them as referring to “Specific terms”, “Related terms”, or “Others”.
  “Specific terms” are those TSRSC terms that can be used to retrieve stem cell research literature and include keywords such as “stem cells” and “nuclear reprogramming.” These terms were derived from the judgment of the experts via a word selection process. This process was completed to determine whether the MeSH term itself specifically referred to stem cells, and if so, tracing it to the parent MeSH term to a point where the parent MeSH term was no longer related to stem cells. 18 MeSH terms were obtained using this process.
  “Related terms” referred to those used for fine-grained classification and retrieval, literature analysis, as well as the analysis for the disease type literature retrieval. Thus, for example, in order to explore stem cells in the development of diseases research, MeSH terms such as “neoplasms” and “musculoskeletal diseases” were included in this category.
  The “Others” category included terms that were not in the TSRSC or related terms. These can be reserved for later, fine-tuned retrieval experiments. Therefore, if the subsequent feedback retrieval results, or if retrieval recall precision, is not ideal, then the other MeSH terms can be used to conduct further analysis, taking into consideration whether it can discriminate better when added to TSRSC or related terms.
5. Retrieval experiment. Articles were retrieved using TSRSC, and the recall and precision ratio were subsequently computed. A series of retrieval experiments were conducted, and adding or removing terms from searches optimized the recall and precision ratios for the tested the search terms. The set of search terms was finalized when the search result was satisfied.
6. MeSH term classification. 128 root MeSH terms served as the core sets used to build the stem cell research classification framework (Additional file 1: Table S1), and then categories were added, which was required by the literature analysis. The goal of this procedure is to build a classification system to group these candidate MeSH terms based on anatomy, disease or stem cell types etc., so that they can facilitate literature classification and facilitate easy information access and retrieval. The TSRSC and related terms were both classified into these categories. If a MeSH term was classified into a category, the upper node MeSH terms were considered and classified into the category if possible.
7. Refinement of the TSRSC and the classified thesauri. The precision and recall ratios for both TSRSC and the classified thesauri were computed; the terms set were refined and updated based on the retrieval experiments. Non-MeSH terms from the articles which have not been retrieved were added to the TSRSC (Additional file 2 for non-MeSH terms as TSRSC).
8. Precision and recall experiments. For the TSRSC, the recall ratios were computed using related modified search terms, or TSRSC, to ascertain how many articles out of the total 5527-article sample set could be retrieved. The precision ratios were calculated by randomly selecting 100 articles and having the experts discern whether or not an article is related. This randomized sampling was repeated three times. In the final test, with a TSRSC-assistant search done for accuracy, the precision ratio was calculated based on a random sample of 1000 articles from the retrieval results.
  For the classified thesauri, 300 articles were randomly selected from the retrieval result set of TSRSC. Experts examined the classification of the articles. For example, if it was determined that 240 out of 300 articles belonged to cardiovascular disease, then this classified thesauri set for cardiovascular disease was used for the recall test. This test was repeated three times. The precision ratios were measured by a randomized selection of 100 articles for each classified thesaurus set retrieval result (Table 2). Experts then determined if the articles were relevant to the classified thesaurus set. The random selection of 100 articles for precision was repeated three times.

## Results

### Construction of thesauri specifically referring to stem cells (TSRSC)

A set of MeSH terms was chosen for the TSRSC based on term frequency and artificial discrimination, reducing the number to 15 terms that specifically refer to stem cells, including “Stem Cells,” “Stem Cell Transplantation,” and “Nuclear Reprogramming.” The MeSH terms were then organized into a tree structure. The 15 terms refer to the root node terms (Additional file 2, terms highlighted as red) and there may be a hierarchy of children nodes under the root nodes. “HL-60 Cells,” “U937 Cells” and “K562 Cells” are the hyponyms of TSRSC. However, they also are hyponyms of some non-TSRSC. While these terms are related to the cell lines widely used in labs, they have little to do with stem cells. When searching in PubMed, for example, the search engine will automatically search the hyponyms of the search terms. Therefore, they might bring noise (unrelated search return of literature) in retrieval. These words, therefore, were removed from the TSRSC. In addition, 5 non-MeSH terms were supplemented—“stemness,” “stem cell,” “progenitor cell,” “ipsc,” and “ips cell.” These new terms can improve the recall and precision ratios, especially for newer articles that do not have indexed MeSH terms.

To better illustrate all these search terms, such as TSRSC, non-MeSH, or terms with noise, these terms were visualized (see Additional file 2) and their hierarchical relationships under MeSH. Multiple retrieval experiments were conducted to optimize the TSRSC. As a result, the recall ratio was 97.70 % and the precision ratio was 94.4 % (Table 1).

**Table 1:** Thesauri specifically referring to stem cells (TSRSC) construction and retrieval test

Retrieval test	Strategies	Recall ratio^a^	Precision ratio^b^
1	Only focus on MeSH term search related to Stem Cells	87.84 %	89 %
2	Refine search terms:exclusion of HL-60 Cells, U937 Cells, K562 Cells	87.53 %	95 %
3	Refine search terms: including term search rather than MeSH only	96.39 %	90 %
4	Final set of search terms: exclusion of “Precursor B-Cell Lymphoblastic Leukemia-Lymphoma” OR “Precursor T-Cell Lymphoblastic Leukemia-Lymphoma” OR “Precursor Cell Lymphoblastic Leukemia-Lymphoma”;	97.70 %	94.40 %
	Addition of “stemness” OR “stem cell” OR “ipsc” OR “ips cell” OR “progenitor cell”		

^a^Recall ratio: Investigate how many articles in the sample set are retrieved using TSRSC (the sample set contains 5527 articles)

^b^Precision ratio: Randomly select 100 literatures retrieved by TSRSC, examine whether the literature is in the field of stem cells. Process repeated three times

For the convenience of future studies, the following nesting search query that contained all the terms in TSRSC can be used by scientists when they conduct a logical search query for stem cell-related articles in PubMed. The query contains multiple OR and NOT operators that connect with other search terms that have been tested for their quality in precision and recall ratios:

**Search query** *=* (*“Stem Cells”***OR***“Stem Cell Transplantation”***OR***“Nuclear Reprogramming”***OR***“Hematopoiesis”***OR***“Stem Cell Niche”***OR***“Bone Marrow Transplantation”***OR***“Adipogenesis”***OR***“Cell Transdifferentiation”***OR***“Stem Cell Research”***OR***“Hematopoietic Stem Cell Mobilization”***OR***“Nuclear Transfer Techniques”***OR***“Cell Dedifferentiation”***OR***“Stem Cell Factor”***OR***“Blastocyst Inner Cell Mass”***OR***“Tumor Stem Cell Assay”***OR***“stemness”***OR***“stem cell”***OR***“progenitor cell”***OR***“ipsc”***OR***“ips cell”*) **NOT***(“HL-60 Cells”***OR***“U937 Cells”***OR***“K562 Cells”)*

The retrieval results of searching “stem cell,” or using the TSRSC terms in PubMed, were compared. Using the search term “stem cell” obtained 114,390 articles (2002/01/01 ~ 2011/12/31). However, using the TSRSC search, it yielded 131,960 articles (2002/01/01 ~ 2011/12/31). An additional data mining algorithm for text clustering was used to further validate the retrieval results. Five hundred articles were randomly selected from each of the retrieval results and the MeSH terms were extracted. Then a literature vector space model was built using R. For example, if it is assumed having two articles, x(t) and y(t), then MeSH terms as topics for article x are T_1_, T_2_, T_3_, T_4_, and MeSH terms for article *y* are T_2_, T_4_, T_5_, T_6_, T_7,_ respectively. Let T represent the union set of the MeSH terms as the topics for both article *x* and *y*. When t has its value as T_1_, T_2_, T_3,_ T_4_, then x(t) = 1, otherwise x(t) = 0; when t has its value as T_2,_T_4,_T_5,_T_6,_T_7_ then y(t) = 1,otherwise y(t) = 0. Then the matrix of article x(t) and y(t) is showed as the following:

**Table:** 

T	T_1_	T_2_	T_3_	T_4_	T_5_	T_6_	T_7_
x(t)	1	1	1	1	0	0	0
y(t)	0	1	0	1	1	1	1

The average article similarity as cosine (x,y) was calculated to cluster the articles (Eq. 1). The clustering is based on the hypothesis that the article similarity is larger when the value of cosine (x, y) is bigger [24]. The result indicated that the article similarity of “stem cell” is 0.8188 versus a TSRSC of 0.8423. The text clustering result shows that the TSRSC, despite using more words, makes the literature cluster closer rather than more dispersed. This supports our hypothesis that there is a significant correlation in the TSRSC words. This relationship makes retrieval using the TSRSC more accurate.

1 $$ \cos \left(x(t),y(t)\right)=\frac{{\displaystyle \sum_{t\in T}x(t)y(t)}}{\sqrt{{\displaystyle \sum_{t\in T}\;x{(t)}^2\cdot }{\displaystyle \sum_{t\in T}\;y{(t)}^2}}} $$

### Classified thesaurus for knowledge organization

In Table 2, it is demonstrated how MeSH terms can be used to build thesaurus (MeSH)-based classification systems for stem cell-based knowledge organization, such as for anatomy. Examples for stem cell-based knowledge organization systems for anatomy, stem cell types, disease related, and stem cell related can be found in Additional file 1. Within each category, a set of MeSH terms can be used for a retrieval task to obtain literature related to a specific category (Additional file 1). Retrieval experiments were conducted to measure the optimization of the classified thesaurus. The recall ratio ranged from 80.5 to 100.0 %, and the precision ratio ranged from 82 to 100 %. In addition to the recall ratio for all selected journals, a recall ratio for the special journal sample was performed. For this, 50 specialized journals for cancer were randomly chosen and then the neoplasm category thesaurus was used to perform a retrieval test. The recall ratios of the cancer journals were then compared. Since all of the categories do not have a devoted special journal, only a few categories were tested for the recall ratios within cancer journals. Even though this method uses more samples, the results are similar to the general recall ratio mentioned above.

**Table 2:** Classified thesauri set for anatomy (partial)

	Category	Thesauri set
1	Cardiovascular System	Cardiovascular System; Cardiovascular Physiological Phenomena; Diagnostic Techniques, Cardiovascular; Cardiovascular Diseases
2	Respiratory System	Respiratory System; Respiratory Physiological Phenomena; Respiratory Tract Diseases; Carcinoma, Lewis Lung; Diagnostic Techniques; Respiratory System
3	Digestive System	Digestive System; Liver Regeneration; Digestive System and Oral Physiological Phenomena; Stomatognathic System; Digestive System Diseases; Diagnostic Techniques; Digestive System; Stomatognathic Diseases; Odontogenic Tumors

High recall and precision ratios were obtained in every category except for adult stem cells in Table 3. We found that the term “adult stem cells” was indexed incompletely in PubMed. Many articles regarding this term were indexed as “stem cells” instead of “adult stem cells.” In addition, there were no “child” node terms under the term “adult stem cells.” Thus, the recall ratio was low. To improve the recall ratio, additional search terms were added, such as “hematopoietic stem cells,” “hematopoietic stem cell transplantation,” and “neural stem cells,” to the category of “adult stem cells”. These additional search terms were found to be more specific and feasible in order to yield more search returns of relevant literature. Through this, the recall ratio was increased to 62.7 %, which importantly indicated that scientists prefer certain terms than standardized MeSH terms to describe a scientific topic. Since there are numerous types of adult stem cells, further investigation and expert consultation may be needed and new, non-MeSH terms should be considered to enrich the TSRSC, such as “bone marrow-derived mesenchymal stromal cells,” “mesenchymal stromal cells from bone marrow,” and “MSCs from bone marrow.” In addition, it is important to note that the categories themselves are crossed, so a MeSH term may belong to more than one category. However, this phenomenon does not affect the accuracy of literature retrieval.

**Table 3:** Retrieval result of classified thesaurus set for anatomy (partial)

Category	Recall ratio^a^	Precision ratio^b^	Recall ratio (special journal sample)
Cardiovascular System	92 %	93.3 %	92.4 %
Respiratory System	88 %	91.6 %	100 %
Digestive System	90 %	100.0 %	89.2 %
Neuro-Sensory System	89 %	100.0 %	96.3 %
Musculoskeletal and Integumentary System	87 %	94.1 %	86.6 %
Urogenital System	92 %	100 %	90.3 %
Hemic and Immune Systems	82 %	100 %	93.8 %
Cardiovascular Diseases	97 %	94.1 %	
Respiratory Tract Diseases	94 %	80.5 %	
Digestive System Diseases	92 %	88.6 %	
Nervous System Diseases	89 %	93.7 %	
Eye Diseases	96 %	100.0 %	
Musculoskeletal Diseases	97 %	85.0 %	
Integumentary System Diseases	93 %	89.1 %	
Urogenital System Diseases	90 %	92.3 %	
Hemic and Immune Systems Diseases	90 %	93.7 %	
Endocrine System Diseases	94 %	92.6 %	
Nutritional and Metabolic Diseases	88 %	87.8 %	
Congenital, Hereditary, and Neonatal Diseases and Abnormalities	89 %	90.0 %	
Neoplasms	84 %	93.0 %	91.7 %
Graft vs Host Disease	84 %	88.8 %	
Virus Diseases	99 %	83.3 %	
Wounds and Injuries	98 %	87.5 %	
Embryonic Stem Cells	99 %	100.0 %	
Adult Stem Cells	94 %	62.7 %	
Neoplastic Stem Cells	94 %	100.0 %	
Induced Pluripotent Stem Cells	99 %	100.0 %	
Reprogramming	96 %	96.6 %	
Stem Cell Transplantation	100 %	94.0 %	
Ethics	96 %	100.0 %	

^a^Recall ratio: Investigates how many articles are retrieved using classified thesauri (Randomly select 300 articles from 5527 and classify them into different categories as benchmarks.)

^b^Precision ratio: Randomly select 100 articles retrieved by classified thesauri and examine whether they belongs to the category

## Discussion

In this study, the procedure of constructing a classified thesaurus was explored and validated through precision and recall ratio tests of combinatory literature search queries with selected MeSH based terms, understanding these terms’ hierarchy relationships, and grouping MeSH terms into different stem cell based knowledge organization systems. The principles of construction were: 1) The MeSH terms were classified based on high/low frequency; 2) Reject terms with too general concept and terms of some substances, such as protein, DNA sequence and molecule; 3) Expert judgement for term selections; and 4) Retrieval experiment.

The validity of the TSRSC was tested by a Vector Space Model. The results of this portion of the study supported the hypothesis that there is significant correlation between the TSRSC terms and retrieval results. Careful selection of a set of appropriated TSRSC terms provided thorough and precise search results. The retrieval results proved to yield both high recall and precision ratios.

In the future, a classified thesaurus for other fields can be constructed following this method. This method can be enhanced by automation, in which artificial intelligence programs can pre-filter some redundant or unrelated terms, such as protein and DNA sequence, to reduce the judgment-work of experts. Data mining programs can also be used to count the MeSH term frequencies, which would include root nodes. In this way, the position of the terms can be quickly ascertained. Furthermore, the MeSH terms can be further classified into a subcategory to provide a further break-down of knowledge organization.

MeSH is an authoritative controlled vocabulary that is highly effective for bibliographic retrieval for systems that use it. However, studies and literature reviews on inter-indexer consistency often find variability and inconsistency, with consistency rates in the 20–40% range [25–27]. Our method takes this into consideration and, in part, addresses this problem to enhance term specificity by looking at how these terms posit in the subcategories within the thesaurus. This is helpful for improving retrieval results of the MeSH terms, which are indexed incompletely. If these selected MeSH terms and the process of stem cell-based knowledge reorganization can be embedded into a retrieval system, the classified thesaurus could support literature category navigation and automatic classification, further achieving automated literature analysis.

Using stem cell research literature as a case study, it was possible to research topic-based knowledge organization schemes, refine, and extend the utilization of MeSH terms and the hierarchical structures for organized stem cell research. This method also facilitates classification-based literature navigation, literature auto-classification, and benefiting knowledge discovery and mining, and scientific data analysis.

The classification of stem cell research literature is more than just simply cross-walk between classification schemes (e.g., Dewey Decimal System) or thesauri (e.g., MeSH), otherwise some meaningful information may be missed if unmatched between two schemes. Upper class term matching may only yield a proximity which may not be precise enough. Thus, the fine-gained classification method proposed in this study provides a new way to construct classification schemes, thus enhancing clarity. The study provided new potentials to possibly merge different classification schemes or metadata connecting with different retrieval systems. It also proposed possible unified retrieval solutions, then obtain related literature across different databases or information repositories. Better than the cross-walk approach between two schemas that might have the potential to miss important information, our approach can evaluate the search term selection efficiency in precision and recall, as well as provide an assessment of their positions and relationships within the MeSH hierarchy structure. The build-up of combinational searchable query using selected MeSH terms could improve literature retrieval and knowledge organization regardless of the complexity of the scientific subject, even it is interdisciplinary across several fields.

The research findings suggested that the TSRSC query given is useful to improve the literature retrieval in stem cell research, but replication of the methodology is required for new queries in other scientific domains as the query is specific to the scientific domain of stem cell research. In order to foster query creation and methodology in other domains, information professionals should advise researchers that the expert steps require significant time and resource investment in order to achieve quality results.

## Conclusions

This study demonstrated a new approach that can mechanically identify and refine a set of MeSH terms to facilitate literature retrieval and knowledge organization in a particular scientific domain. The study also identified non-MeSH terms that were valuable for improving precision and recall for literature retrieval and therefore enrich the descriptions of a dynamic and interdisciplinary research area such as stem cell research. Based on the reconstructed thesaurus created from these selected terms, the study indicated that identifying a proper set of MeSH terms, and their alternatives, improves the precision and recall ratios of the literature retrieval. Through a case study of stem cell research, we explored a way to construct a classified thesaurus, thus offering one possible solution to the problem of literature retrieval. This classified thesaurus results from a knowledge reconstruction that categorizes existing thesauri and ultimately improves the retrieval of clustered literature. With experiment-proven efficacy, this exploration lays the foundation for future work–automatic literature classification and navigation which may serve science and technology management. This approach can guide scientists, medical researchers, and librarians to use MeSH, or other tools, to construct a set of domain-specific search terms for literature research; simultaneously, this study will allow information professionals to develop a MeSH-based classified thesaurus for knowledge management in regards to a particular research topic. The approach outlined in this study can optimize retrieval performance and knowledge organization by selecting the right MeSH terms when dealing with a dynamic and complex scientific research topic for scientists.

## Additional files

Additional file 1: Stem cell related thesaurus for knowledge reconstruction. (DOCX 31 kb) Additional file 2: Visualization of stem cell related search terms (MeSH or Non-MeSH). (EPS 18513 kb) Additional file 3: Stem cell research journal list and collected MeSH terms with frequencies. (XLSX 98 kb)

## Abbreviations

KOS: knowledge organization system
MeSH: medical subject headings
TSRSC: thesaurus specifically referring to stem cells

## References

[1] Wang ZH (2009). Developments and trends in KOS. Libr Inf Sci.

[2] Xia GH, Li JL, Li DY (2011). Design and realization of medical literature intelligence retrieval system based on MeSH. J Med Inform.

[3] Liu HM, Hou HQ (2006). Research progresses of interactive operation of information searching language in the near 10-year. Libr Theory Pract.

[4] Falagas ME, Pitsouni EI, Malietzis GA (2008). Comparison of PubMed, Scopus, Web of Science, and Google Scholar: Strengths and weaknesses. FASEB J.

[5] Rajpal DK, Kumar V, Agarwal P (2011). Scientific literature mining for drug discovery: a case study on obesity. Drug Dev Res.

[6] Aljaber B, Martinez D, Stokes N (2011). Improving MeSH classification of biomedical articles using citation contexts. J Biomed Inform.

[7] Cimino JJ (1998). Desiderata for controlled medical vocabularies in the twenty-first century. Methods Inf Med.

[8] Coletti MH, Bleich HL (2001). Medical subject headings used to search the biomedical literature. J Am Med Inform Assoc.

[9] Mancini F, Sousa FS, Teixeira FO (2011). Use of Medical Subject Headings (MeSH) in Portuguese for categorizing web-based healthcare content. J Biomed Inform.

[10] Zhang XC, Huang DS, Li F (2011). Cancer nursing research output and topics in the first decade of the 21st century: Results of a bibliometric and coword cluster analysis. Asian Pac J Cancer Prev.

[11] Zhang H, Han D, Bai X (2011). Application of genetic algorithm to identify hot topics from medical literature. New Technol Libr Inf Serv.

[12] Yang Y, Cui L (2011). Evolution of topics about medical informatics by improved co-word cluster analysis. New Technol Libr Inf Serv.

[13] Yan QL, Zhang Y (2001). Review of medical subject headings (MeSH). J Inf.

[14] Sánchez D, Solé-Ribalta A, Batet M (2012). Enabling semantic similarity estimation across multiple ontologies: an evaluation in the biomedical domain. J Biomed Inform.

[15] Yetisgen-Yildiz M, Pratt W (2009). A new evaluation methodology for literature-based discovery systems. J Biomed Inform.

[16] Wilczynski N L, Walker C J, McKibbon K A, et al. Reasons for the loss of sensitivity and specificity of methodologic MeSH terms and textwords in MEDLINE. In Proceedings of the Annual Symposium on Computer Application in Medical Care. Philadelphia: American Medical Informatics Association. 1995;436. PMC2579130 8563319

[17] Fu LD, Aphinyanaphongs Y, Wang L (2011). A comparison of evaluation metrics for biomedical journals, articles, and websites in terms of sensitivity to topic. J Biomed Inform.

[18] Stevenson M, Guo Y (2010). Disambiguation in the biomedical domain: the role of ambiguity type. J Biomed Inform.

[19] Huang M, Névéol A, Lu Z (2011). Recommending MeSH terms for annotating biomedical articles. J Am Med Inform Assoc.

[20] Ruau D, Mbagwu M, Dudley JT (2011). Comparison of automated and human assignment of MeSH terms on publicly-available molecular datasets. J Biomed Inform.

[21] Maccalli C, De Maria R (2015). Cancer stem cells: perspectives for therapeutic targeting. Cancer Immunol Immunother.

[22] Uccelli A, Prockop DJ (2010). Why should mesenchymal stem cells (MSCs) cure autoimmune diseases?. Curr Opin Immunol.

[23] Ebert AD, Svendsen CN (2010). Human stem cells and drug screening: opportunities and challenges. Nat Rev Drug Discov.

[24] Yao QY (2008). Research of VSM-based Chinese text clustering algorithms.

[25] Hooper RS (1965). Indexer Consistency Tests: Origin, Measurement, Results, and Utilization.

[26] Medelyan O, Witten IH (2008). Domain‐independent automatic keyphrase indexing with small training sets. J Am Soc Inf Sci Technol.

[27] Vaughan Hughes A, Rafferty P (2011). Inter-indexer consistency in graphic materials indexing at the National Library of Wales. J Doc.

