# Supplementary material for: Reengineering of MeSH thesauri for term selection to optimize literature retrieval and knowledge reconstruction in support of stem cell research
Source: BMC Med Inform Decis Mak. 2016 May 23;16:54. doi: 10.1186/s12911-016-0298-z (PMC4878086; doi:10.1186/s12911-016-0298-z)
Supplement: Additional file 1: — Stem cell related thesaurus for knowledge reconstruction. (DOCX 31 kb) [file 12911_2016_298_MOESM1_ESM.docx]

**Table S1 Categories of Anatomy**

| **No.** | **Categories** | **Thesauruses Set** |
| --- | --- | --- |
| 1 | Cardiovascular System | Cardiovascular System；Cardiovascular Physiological Phenomena； Diagnostic Techniques, Cardiovascular；Cardiovascular Diseases |
| 2 | Respiratory System | Respiratory System；Respiratory Physiological Phenomena；  Respiratory Tract Diseases；Carcinoma, Lewis Lung；Diagnostic Techniques, Respiratory System |
| 3 | Digestive System | Digestive System；Liver Regeneration；Digestive System and Oral Physiological Phenomena；Stomatognathic System； Digestive System Diseases；Diagnostic Techniques, Digestive System；  Stomatognathic Diseases；Odontogenic Tumors |
| 4 | Neuro-Sensory System | Nervous System；Sense Organs；Nervous System Physiological Phenomena；Neurogenesis；Nerve Regeneration；Spinal Cord Regeneration；Nose；ear；Eye；Nose Diseases；Ear Diseases；Eye Diseases；Nervous System Diseases；Brain Tissue Transplantation； Nerve Degeneration；Gliosis；Neurosurgical Procedures； Neuroectodermal Tumors；Nervous System Neoplasms；Diagnostic Techniques, Otological；Diagnostic Techniques, Neurological； Neuroectodermal Tumors；Neoplasms, Nerve Tissue；Neuroendocrine  Tumors |
| 5 | Musculoskeletal and Integumentary System | Musculoskeletal System；Integumentary System；Connective Tissue； Bone Regeneration；Chondrocytes；Keratinocytes；Musculoskeletal and Neural Physiological Phenomena；Osteoblasts；Myocytes, Smooth Muscle；Muscle Cells； Basement Membrane；Skin Physiological Phenomena；Odontoblasts；Integumentary System Physiological Phenomena；Musculoskeletal Physiological Phenomena； Musculoskeletal Diseases；Amyotrophic Lateral Sclerosis；Muscular  Dystrophy, Animal；Tendon Injuries；Fracture Healing；Knee Injuries； |

| Ossification, Heterotopic；Neoplasms, Bone Tissue； Chondroblastoma；Chondroma；Chondrosarcoma；Skin Diseases； Neoplasms, Adnexal and Skin Appendage； Neoplasms, Ductal,  Lobular, and Medullary；Neoplasms, Fibroepithelial | | |
| --- | --- | --- |
| 6 | Urogenital System | Urogenital System；Reproductive and Urinary Physiological Phenomena；Reproductive Techniques；Urothelium；Female Urogenital Diseases；Male Urogenital Diseases；Diagnostic  Techniques, Urological；Germinoma；Mesonephroma |
| 7 | Hemic and Immune Systems | Hematopoietic System；Blood Banks；Immune System；Immunologic Factors；Immune System Phenomena；Immunologic Techniques； Immune System Diseases；Immunotherapy；Hematologic Diseases； Leukemia；Lymphatic Vessel Tumors；Lymphoma；Multiple Myeloma；Plasmacytoma；Waldenstrom Macroglobulinemia；  Hematopoietic Stem Cell Transplantation |

**Table S2 Categories of Disease**

| **No.** | **Categories** | **Thesauruses Set** |
| --- | --- | --- |
| 1 | Cardiovascular Diseases | Cardiovascular Diseases |
| 2 | Respiratory Tract Diseases | Respiratory Tract Diseases |
| 3 | Digestive System Diseases | Digestive System Diseases；Stomatognathic Diseases； Odontogenic Tumors |
| 4 | Nervous System Diseases | Nervous System Diseases；Nerve Degeneration；Gliosis； Neurosurgical Procedures；Neuroectodermal Tumors； Nervous System Neoplasms； Neuroectodermal Tumors；  Neoplasms, Nerve Tissue；Neuroendocrine Tumors |
| 5 | Eye Diseases | Eye Diseases |

| 6 | Musculoskeletal Diseases | Musculoskeletal Diseases；Muscular Dystrophy, Animal； Ossification, Heterotopic；Neoplasms, Bone Tissue； Chondroblastoma；Chondroma；Chondrosarcoma |
| --- | --- | --- |
| 7 | Integumentary System Diseases | Skin Diseases；Neoplasms, Adnexal and Skin Appendage； Neoplasms, Ductal, Lobular, and Medullary；Neoplasms, Fibroepithelial |
| 8 | Urogenital System Diseases | Female Urogenital Diseases；Male Urogenital Diseases；  Diagnostic Techniques, Urological；Germinoma； Mesonephroma |
| 9 | Hemic and Immune Systems Diseases | Immune System Diseases；Immunotherapy；Hematologic Diseases；Leukemia；Lymphatic Vessel Tumors； Lymphoma；Multiple Myeloma；Plasmacytoma；  Waldenstrom Macroglobulinemia |
| 10 | Endocrine System Diseases | Endocrine System Diseases；Neuroendocrine Tumors |
| 11 | Nutritional and Metabolic  Diseases | Nutritional and Metabolic Diseases |
| 12 | Congenital, Hereditary, and Neonatal Diseases and  Abnormalities | Congenital, Hereditary, and Neonatal Diseases and Abnormalities |
| 13 | Neoplasms | Neoplasms；Gene Expression Regulation, Neoplastic； Antineoplastic Agents ； Neoplasm Transplantation ； Neoplastic Processes；Drug Resistance, Neoplasm；Tumor Microenvironment ； Tumor Burden ； Antineoplastic Protocols；Drug Screening Assays, Antitumor；Oncolytic Virotherapy； |
| 14 | Graft vs Host Disease | Graft vs Host Disease；Transplantation Immunology；  Transplantation Tolerance；Histocompatibility Testing； |
| 15 | Virus Diseases | Virus Diseases |
| 16 | Wounds and Injuries | Wounds and Injuries；Wound Healing |

**Table S3 Categories of Stem Cells**

| **No.** | **Categories** | **Thesauruses Set** |
| --- | --- | --- |
| 1 | Embryonic Stem Cells | Embryonic Stem Cells |
| 2 | Adult Stem Cells | Adult Stem Cells；Peripheral Blood Stem Cell Transplantation；Bone Marrow Transplantation； Hematopoietic Stem Cell Mobilization Hematopoietic Stem Cells；Hematopoietic Stem Cell  Transplantation；Hematopoietic Stem Cell Mobilization； Neural Stem Cells；  Peripheral Blood Stem Cell Transplantation  Bone Marrow Transplantation；bone marrow-derived mesenchymal stromal cells；mesenchymal stromal cells from bone marrow；MSCs from bone marrow |
| 3 | Neoplastic Stem Cells | Neoplastic Stem Cells；Tumor Stem Cell Assay； |
| 4 | Induced Pluripotent Stem  Cells | Induced Pluripotent Stem Cells； ipsc ；ips cell |

**Table S4 Other Categories**

| **No.** | **Categories** | **Thesauruses Set** |
| --- | --- | --- |
| **1** | Reprogramming | Cell Transdifferentiation；Cell Dedifferentiation；Nuclear  Reprogramming；Induced Pluripotent Stem Cells |
| **2** | Stem Cell  Transplantation | Stem Cell Transplantation；Bone Marrow Transplantation |
| **3** | Ethics | Bioethics；Ethics, Research；Ethics, Medical；Stem Cell Transplantation/ethics；Nuclear Reprogramming/ethics；Bone Marrow Transplantation/ethics；Stem Cell Research/ethics； Hematopoietic Stem Cell Mobilization/ethics；Nuclear Transfer  Techniques/ethics |
